# Supplementary figures and images for: Genome-Wide Identification of DOF Gene Family and the Mechanism Dissection of SbDof21 Regulating Starch Biosynthesis in Sorghum
Source: Int J Mol Sci. 2022 Oct 12;23(20):12152. doi: 10.3390/ijms232012152 (PMC9603474; doi:10.3390/ijms232012152)

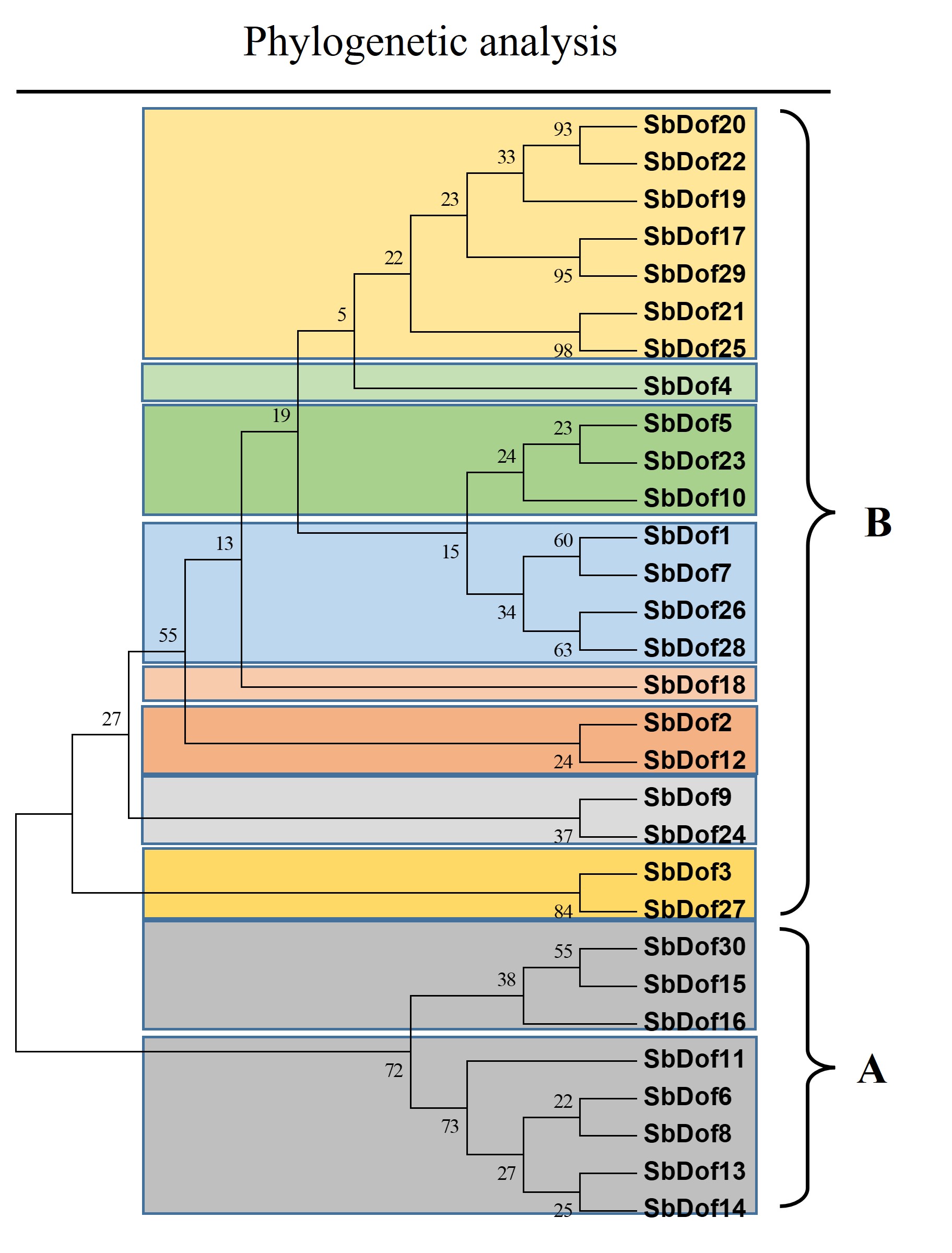

Supplement: Supplementary file 1 [file ijms-23-12152-s001.zip › Figure S1=Revised.jpg]

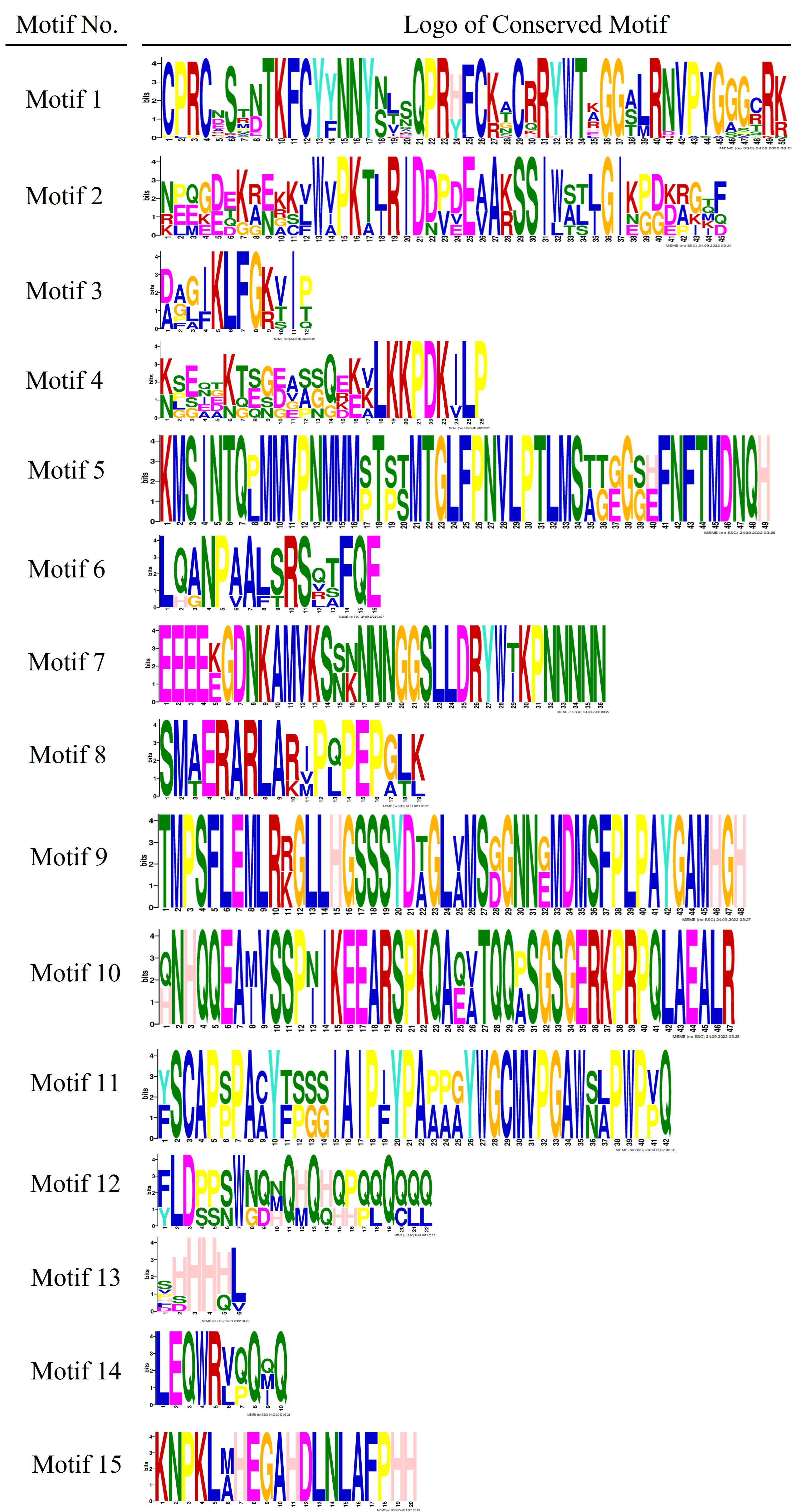

Supplement: Supplementary file 1 [file ijms-23-12152-s001.zip › Figure S2=Revised.jpg]

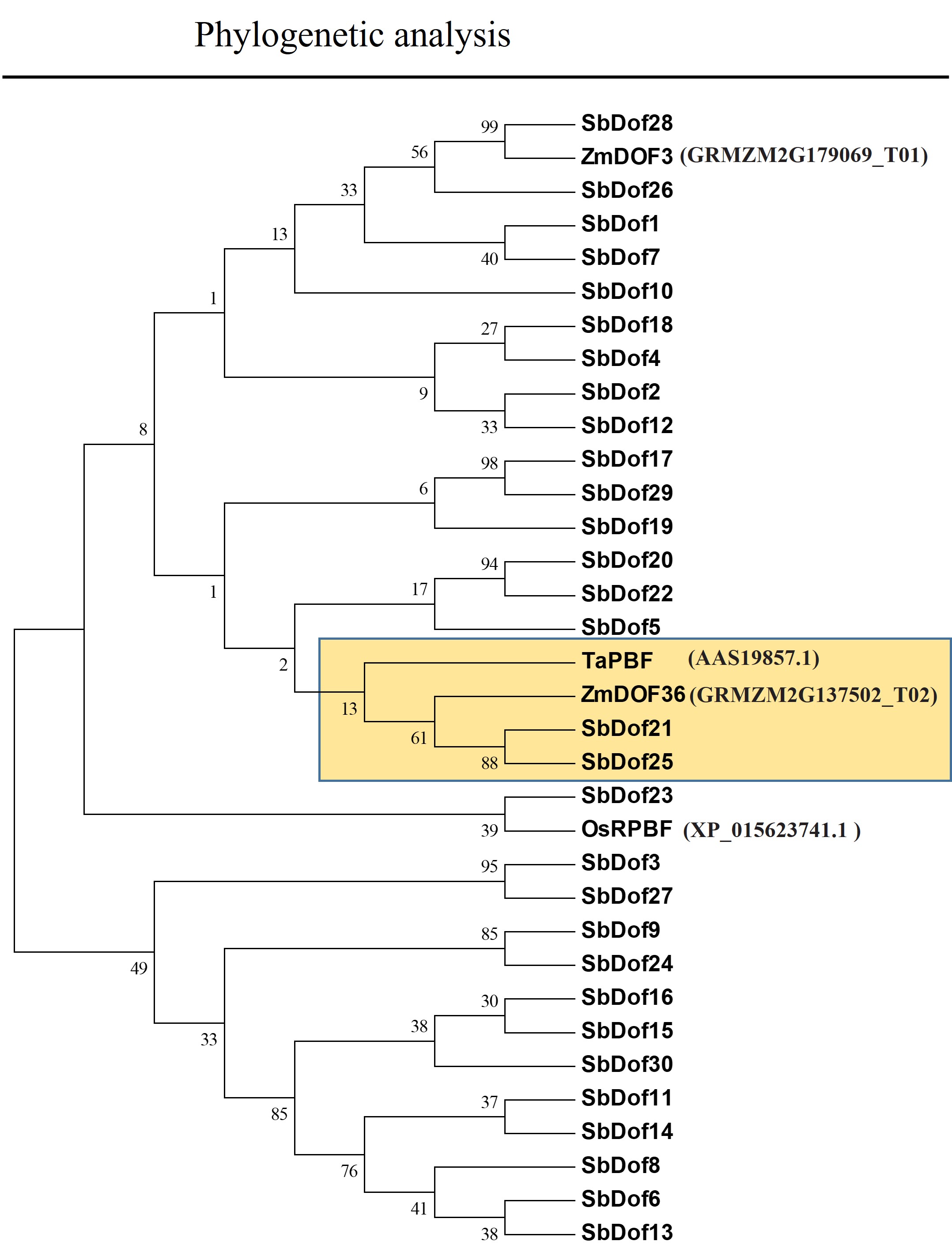

Supplement: Supplementary file 1 [file ijms-23-12152-s001.zip › Figure S3=Revised.jpg]
